# Impact of Ex Vivo Bisphenol A Exposure on Gut Microbiota Dysbiosis and Its Association with Childhood Obesity

**DOI:** 10.3390/jox15010014

**Published:** 2025-01-17

**Authors:** Gracia Luque, Pilar Ortiz, Alfonso Torres-Sánchez, Alicia Ruiz-Rodríguez, Ana López-Moreno, Margarita Aguilera

**Affiliations:** 1 Human Microbiota Laboratory, Institute of Nutrition and Food Technology “José Mataix” (INYTA), Centre of Biomedical Research, University of Granada, 18016 Granada, Spain; gracialuque@ugr.es (G.L.); piortiz@ugr.es (P.O.); alfonsotorress@correo.ugr.es (A.T.-S.); aliruizrodriguez@ugr.es (A.R.-R.); maguiler@ugr.es (M.A.); 2 Department of Microbiology, Faculty of Pharmacy, University of Granada, Campus of Cartuja, 18071 Granada, Spain; 3 IBS: Instituto de Investigación Biosanitaria, 18012 Granada, Spain

**Keywords:** ex vivo BPA exposure, microbiota taxa, dysbiosis, obesity

## Abstract

Dietary exposure to the plasticiser bisphenol A (BPA), an obesogenic and endocrine disruptor from plastic and epoxy resin industries, remains prevalent despite regulatory restriction and food safety efforts. BPA can be accumulated in humans and animals, potentially exerting differential health effects based on individual metabolic capacity. This pilot study examines the impact of direct ex vivo BPA exposure on the gut microbiota of obese and normal-weight children, using 16S rRNA amplicon sequencing and anaerobic culturing combined methods. Results showed that direct xenobiotic exposure induced modifications in microbial taxa relative abundance, community structure, and diversity. Specifically, BPA reduced the abundance of bacteria belonging to the phylum *Bacteroidota*, while taxa from the phylum *Actinomycetota* were promoted. Consistently, *Bacteroides* species were classified as sensitive to BPA, whereas bacteria belonging to the class *Clostridia* were identified as resistant to BPA in our culturomics analysis. Some of the altered bacterial abundance patterns were common for both the BPA-exposed groups and the obese non-exposed group in our pilot study. These findings were also corroborated in a larger cohort of children. Future research will be essential to evaluate these microbial taxa as potential biomarkers for biomonitoring the effect of BPA and its role as an obesogenic substance in children.

## 1. Introduction

Dietary xenobiotics or artificial compounds, including synthetic chemicals such as packaging plastics, flavourings and additives, pesticides and other environmental pollutants, can interact with the body when ingested through food, beverages, and medications. Therefore, cumulative human exposure to dietary xenobiotics is almost inevitable [1]. Nowadays, there is a special interest in plastic-derived compounds with obesogenic effects, such as bisphenols, particularly with regard to exposure during the early life stages [2]. Bisphenol A (BPA) has been detected in various biological fluids, including the serum, urine, saliva, and blood, as well as in adipose tissue, the liver, and the placenta [3,4]. Due to its obesogenic [5] and endocrine-disrupting effects [6,7,8] along with growing evidence of the health risks associated with BPA exposure, authorities have progressively lowered the recommended maximum exposure limits [9].

Xenobiotic compounds are metabolised differently by the host’s endogenous metabolism and may not be completely eliminated, allowing their accumulation in tissues and organs [5]. Despite significant advancements in BPA biomonitoring, particularly in understanding its metabolisation, bioaccumulation, and excretion processes, these mechanisms are still not fully understood [10]. Limitations have been identified in urine-based BPA measurements, primarily due to the compound’s rapid metabolism [11], which may not accurately reflect long-term exposure compared to other biospecimens, such as nails or hair [12]. These specimens provide a better representation of long-term BPA exposure. Moreover, our previous findings highlight the important role of the microbiota in BPA metabolism, shedding light on the emerging field of BPA pharmacokinetics and toxicomicrobiomics [13].

At the intestinal level, dietary xenobiotics influence gut microbiota [14]. Alterations in the gut microbiota due to xenobiotic exposure can contribute to health disorders. Common outcomes of microbial imbalance, known as dysbiosis, include inflammation, oxidative stress, and both intestinal and metabolic issues, which in turn increase the risk of developing chronic non-communicable diseases such as obesity. Understanding the composition, resilience, balance, and diversity of the gut microbiota is essential for evaluating its role in host health. Furthermore, it is necessary to know whether the microbiota is more or less sensitive to xenobiotic exposure, which can potentially be associated with the inhibition or overgrowth of specific taxa and their association with a disease-linked dysbiotic microbiota [15].

Additionally, certain microorganisms from the gut microbiota possess enzymatic capabilities that enable the degradation or transformation of xenobiotics into harmful or harmless compounds. Identifying microbial taxa with detoxification capacities is thus critical, not only for advancing bioremediation strategies but also for exploring potential probiotic applications [16,17]. This emerging field of research, termed toxicomicrobiomics, underscores the importance of discovering taxa with xenobiotic biotransformation potential.

Specifically, we focus on the obesogenic potential of BPA and its impact on the microbial community, particularly its association with a dysbiotic microbiota pattern, observed in children with obesity [18]. This pilot study aims to evaluate the direct effect of ex vivo *BPA* exposure on microbiota from normal-weight children and those with obesity, using 16S rRNA amplicon sequencing and culturomics methods, comparing these samples to non-exposed samples to acknowledge restrictions linked to the experimental conditions. Furthermore, comparative analysis with a larger cohort allows to verify the similarity between observed BPA-altered taxa patterns and dysbiosis triggered in childhood obesity.

## 2. Materials and Methods

### 2.1. Microbiota Sampling and BPA Experimental Exposure Processing

A total of 28 faecal samples from Spanish children aged 5 to 11 years were selected from a larger cohort study that complied with ethical requirements and the Declaration of Helsinki [15]. This study was explained to all the parents or legal tutors of participants prior enrolment, and written and signed informed consent was obtained for each child.

Faecal samples from participants were selected according to anthropometric data (Table 1) and 16S rRNA microbiota before treatment. Criteria were applied to establish two well-differentiated clinical/biological populations: the body mass index (BMI) category was established by the World Health Organization (WHO) [19] for those in the normal-weight group (NW, n = 15; median 15.43) and obesity group (OB, n = 13; median 23.47); the age range was established (5–11; median 8 for both females and males); and gender was balanced within the groups. Microbiota 16S rRNA were revised so that we could choose different *Bacillota*/*Bacteroidota* ratios (NW < 0.8–4.5 and OB > 4.5–120).

Stool samples were freshly and anaerobically collected from each participant and maintained frozen at −80 °C until processing [15]. Faecal samples (1 g) were homogenised in PBS (1:10 *w*/*v*) and further exposed to 50 ppm of BPA. We selected the BPA concentration in accordance with experimental work from previous teams after a thorough literature search in order to establish doses and associated pathophysiological and clinical effects [13,15]. Specifically, a 50 ppm concentration served as an approximation of BPA’s potential toxic effects, as initially supported by the TDI (Tolerable Daily Intake), LOAEL (Lowest Observed Adverse Effect Level) and NOAEL (No Observed Adverse Effect Level) from the European Food Safety Authority (EFSA 2015), despite the updates in 2023.

BPA exposure was consistently conducted for 10 days at 37 °C under anaerobic conditions to ensure homogeneous microbial community effects and to evaluate concomitant effects and restrictions linked to timing, solvents, nutrients, and experimental ex vivo conditions. The study design included four groups: (1) non-exposed normal-weight samples (NW), (2) non-exposed obesity samples (OB), (3) BPA-exposed normal-weight samples (NW10), and (4) BPA-exposed obesity samples (OB10).

**Figure A1:**
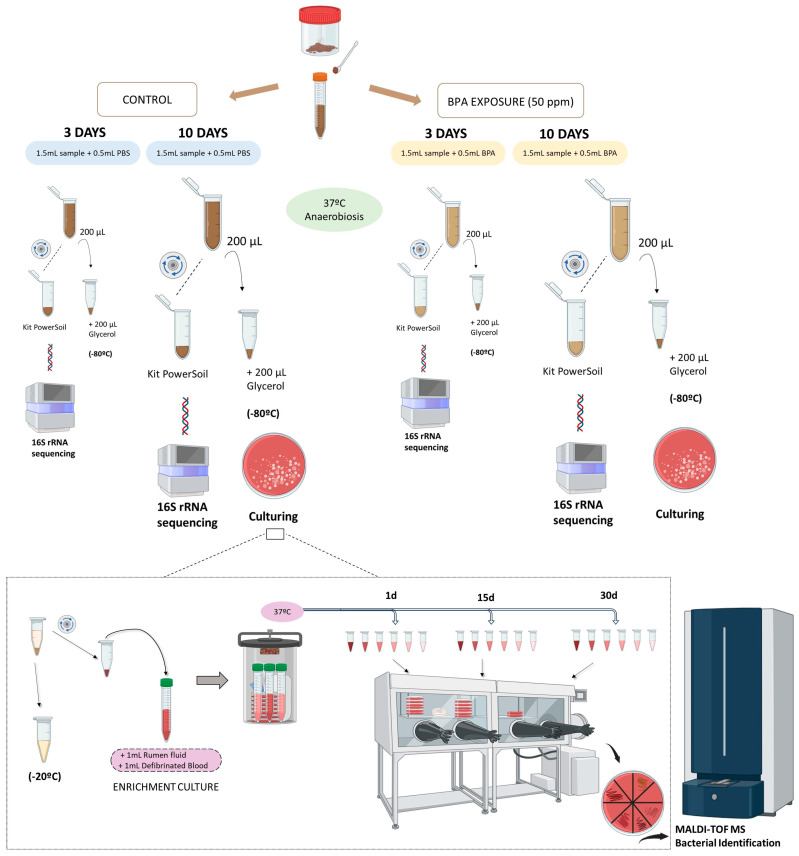
Stool sampling and ex vivo BPA exposure procedure.

Equal aliquots of 200 µL from each exposed and non-exposed sample underwent 16S rRNA amplicon sequencing and culturomic approaches. The detailed procedure is outlined in Figure A1.

### 2.2. DNA Extraction for 16S rRNA Gene Amplicon Library Preparation

Total DNA from faecal samples was extracted using the DNeasy PowerSoil Kit (Qiagen^®^, Hilden, Germany), according to the manufacturer’s instructions. To disrupt the faecal matrix, BioSpec Products Mini-Beadbeater-8 was used. Finally, DNA was eluted in 80 µL of solution, and the DNA concentration was checked using spectrophotometry (biophotometer Eppendorf^®^ D30, Hamburg, Germany). The isolated DNA was sent for external sequencing (Novogene Bioinformatics Technology Co., Ltd., Cambridge, UK) to identify the bacterial taxa and diversity in each sample. A fragment of 466bp containing the V3-V4 region of the prokaryotic small ribosomal subunit 16S rRNA was amplified. For this purpose, the specific forward primer 341F (5′-CCTAYGGGRBGCASCAG-3′) and reverse primer 806R (5′-GGACTACNNGGGTATCTAAT-3′) were used, each linked to a corresponding barcode. PCR products were purified and used to construct a gene library, which was sequenced using the Illumina NovaSeq PE250 platform.

The DADA2 pipeline [20] was used to correct potential sequencing errors and obtain Amplicon Sequence Variants (ASVs). ASVs were taxonomically classified using the “classify-sklearn” algorithm in the QIIME2 platform (https://library.qiime2.org/, accessed on 20 October 2023). ASVs not assigned to bacteria (such as mitochondria or chloroplast) were removed.

### 2.3. Culturomics Approach

For the culturing-based study, faecal samples (both BPA-exposed and non-exposed) were cultured after the addition of pre-enrichment media (sheep blood and sterile fresh rumen fluid in equal parts *v*/*v*, prepared according to the protocol (Appendix B)). Further microbial recovery enrichment was carried out at 37 °C for 1, 15 and 30 days under anaerobic conditions. Subsequently, serial dilutions were spread on Columbia agar with 5% sheep blood medium (MAIM S.L, Barcelona, Spain) and incubated in an anaerobic workstation (Whitley A35 Anaerobic workstation, Don Whitley Scientific Limited, Shipley, UK) for 48 h. Culturomic approach has been improved by Armetta et al. [21] using combined methodologies to select and promote difficult gut bacteria for growth.

A maximum of 16 isolates were selected from each sample applying the picking method [22]. Pure cultures were identified using matrix-assisted laser desorption ionisation–time of flight (MALDI-TOF-Biotyper Sirius-Bruker), and identification of the data was performed using MALDI Biotyper MSP Identification Standard Method 1.1 software from the Hospital Universitario San Cecilio, Granada. If isolates could not be identified using this method, DNA extraction and 16S rRNA gene sequencing using the Sanger method were applied. Sanger sequencing was performed using three universal primers: F357 (5′-CTCCTACGGGAGGCAGCA-3′), R519 (5′-GWATTACCGCGGCKGCTG-3′), and F915 (5′-GGGCCCGCACAAAGCGGTGG-3′).

### 2.4. Statistical Analysis

All analyses were carried out with R version 4.3.2. within RStudio v1.2, primarily with the packages Phyloseq [23], Vegan [24], microbiome [25], microViz [26], and ggplot2 [27]. Bonferroni-adjusted *p* values were generated where appropriate. *p* and Bonferroni-adjusted *p* values of 0.05 were considered significant unless otherwise stated.

Statistical differences in anthropometric continuous variables were evaluated using the Mann–Whitney U test, whereas the gender variable was analysed using the chi-square test. Alpha diversity was estimated by the observed number of ASVs, and the Shannon and Simpson diversity indexes. The statistical significance of the differences in alpha diversity was calculated using the pairwise nonparametric Mann–Whitney U test corrected for multiple comparisons. A nonmetric multidimensional scaling (nMDS) plot based on the Bray–Curtis dissimilarity matrix and PERMANOVA tests were used to visualise and test for the significant differences in the overall microbial community composition between the study groups, respectively.

To compare the microbial composition of communities at the species level and identify patterns in microbial abundance following BPA exposure, the ANCOM-BC2 (Analysis of Compositions of Microbiomes with Bias Correction 2) method was employed [28]. This method included a sensitivity analysis for pseudo-count addition (applied to zero counts before the log transformation) using linear regression models on the bias-corrected log abundance table with various pseudo-counts. To further explore microbiota biomarkers associated with BMI and BPA exposure, the MaAsLin2 statistical model was used with default parameters [29]. This model is designed to identify covariate-associated microbial taxa.

Regarding the anaerobic-culture results, colony-forming units per gram of stools (CFU/g) were determined. The Kruskal–Wallis test was employed to assess differences in CFU/g among groups, accounting for the non-normality of the data. Pairwise comparisons between the study groups (NW, OB, NW10, OB10) were conducted using the Mann–Whitney U test, with *p* values adjusted for multiple comparisons. A phylogenetic tree with 56 unique isolated species was created through the NCBI Common Taxonomy Tree tool and plotted with the R package *ggtree* [30].

Several taxon ratios based on the relative abundance of ASVs were calculated from 150 faecal samples from children belonging to the OBEMISRISK panel (NW = 93, OB = 57), including data published in a previous study [15]. The statistical significance of the differences in ratios was calculated using the Mann–Whitney U test.

## 3. Results

### 3.1. Anthropometric Characteristics of the Pilot Study Population

The median age of the 28 microbiota donors, 12 boys and 16 girls from Spain, was 8. There were no significant differences in gender between the selected samples. We found significant differences in the mean BMI between the study groups (NW, OB) (*p* < 0.001), as expected according to the selection criteria (Table 1).

### 3.2. Changes in Gut Microbiota After Ex Vivo BPA Exposure Determined by 16S rRNA Amplicon Sequencing

#### 3.2.1. Microbiota Composition After BPA Exposure

After quality control, 16S rRNA sequencing was successful for 46 samples (NW (n = 12); O (n = 9); NW10 (n = 13); OB10 (n = 12)). A total of 32,447.660 reads remained for analysis (mean ± SEM, 705,383.91 ± 26,324.99 reads per sample), and a good coverage of 99.99% was obtained. These reads were aligned and grouped into 393 ASVs, representing 94 taxonomic genera from 7 phyla.

**Figure 1:**
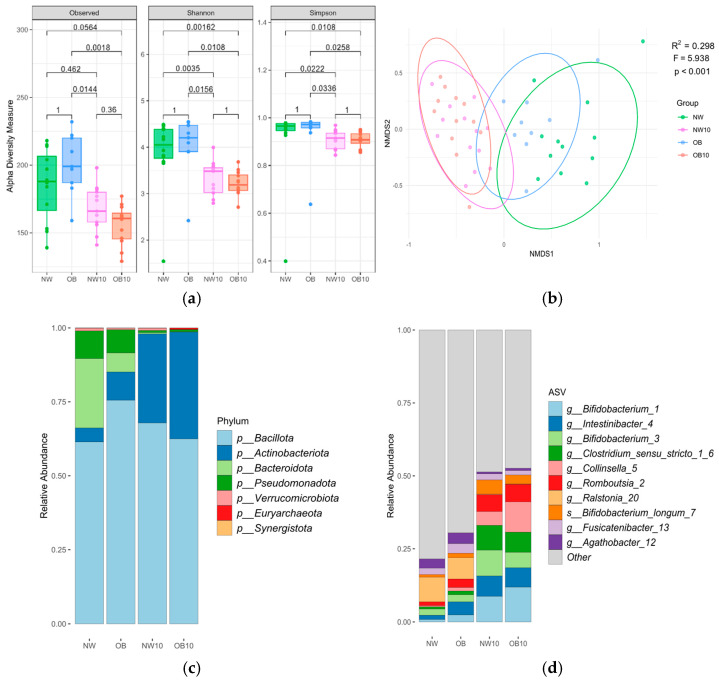
Description of children’s gut microbiota before and after ex vivo BPA exposure according to the study groups, results based on 16S rRNA gene amplicon sequencing. (**a**) Box plots of the alpha diversity indices; (**b**) beta diversity: nMDS plot based on Bray–Curtis distance, with samples as points and ellipses coloured by study groups. PERMANOVA test results (R^2^, F, *p*-value) are indicated in the plot. NW, green; OB, blue; NW10, pink; OB10, orange. Mean relative abundance of indicated phyla (**c**) and ASVs (**d**).

Indices assessing microbial richness and evenness (Figure 1a) revealed changes in microbiota diversity following BPA exposure. Alpha diversity, which was higher in samples from NW and OB children, was significantly reduced after 10 d of BPA exposure in both study groups.

Beta diversity analysis using Bray–Curtis distances (Figure 1b) revealed that the microbiota samples from the NW and OB groups were clustered together and separately from the NW10 and OB10 groups, indicating the strong effect of BPA exposure. Interestingly, the OB group was clustered more closely to the exposed groups (NW10, OB10, R^2^ = 0.225, R^2^ = 0.253) than the NW group (NW10, OB10, R^2^ = 0.295, R^2^ = 0.318, respectively), suggesting that the OB samples were more similar to the BPA-exposed communities (Table A1). Significant differences in microbiota composition were not found either when comparing genders (male, female) or ages.

Figure 1c shows the mean phylum relative abundance, with *Bacillota* being the predominant phylum in all groups. Remarkably, the abundance of *Bacteroidota* showed a drastic reduction, and that of the phylum *Actinomycetota* increased in BPA-exposed groups. At the ASV level (Figure 1d), the BPA-exposed groups (NW10 and OB10) showed an altered microbial composition compared with that of non-exposed groups (NW and OB), with a notable increase in ASVs from the genera *Bifidobacterium*, *Clostridium sensu stricto* group, *Collinsella*, and *Romboutsia*.

**Figure 2:**
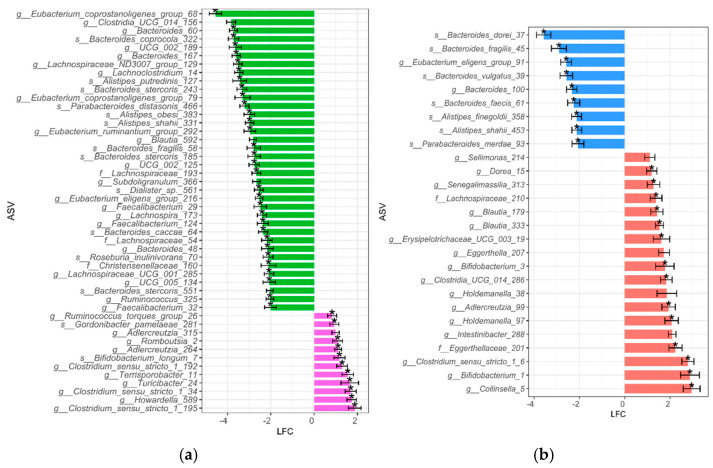
Differentially abundant ASVs between exposed and non-exposed groups. To identify taxa that were differentially abundant due to BPA exposure within each BMI group, ANCOM-BC2 analysis was performed: (**a**) green bars indicate taxa that were significantly more abundant in the NW group, while pink represents taxa that were significantly more abundant in the NW10 group; (**b**) blue bars indicate taxa that were significantly more abundant in the OB group, while red represents taxa that were significantly more abundant in the OB10 group. * features not sensitive to pseudo-count addition. ASVs with a log fold change between—1.99 and 0 are not shown here to simplify plot size.

The differential BPA susceptibility of taxa within each BMI group is represented in Figure 2. We observed that more ASVs were significantly reduced after BPA exposure when comparing NW10 to NW, with ASVs mostly assigned to *Bacteroidales* and *Clostridia* (Figure 2a), in contrast to when we compared the OB10 group to the OB group, which revealed a significant reduction in several ASVs, assigned mostly to genera *Bacteroides*, *Parabacteroides*, *Alistipes*, and *Eubacterium* (Figure 2b).

On the contrary, we found that ASVs were more significantly increased after BPA exposure in the OB10 group compared to in the OB group and in the NW10 group compared to the NW group (Figure 2a,b). In summary, this analysis showed an increase in ASVs classified as *Bifidobacterium*, *Adlercreutzia* and *Clostridium sensu stricto* due to BPA exposure; however, we also observed a differential effect of BPA on each BMI group.

#### 3.2.2. Microbiota Taxa Associated with BPA Exposure and Obesity

**Figure 3:**
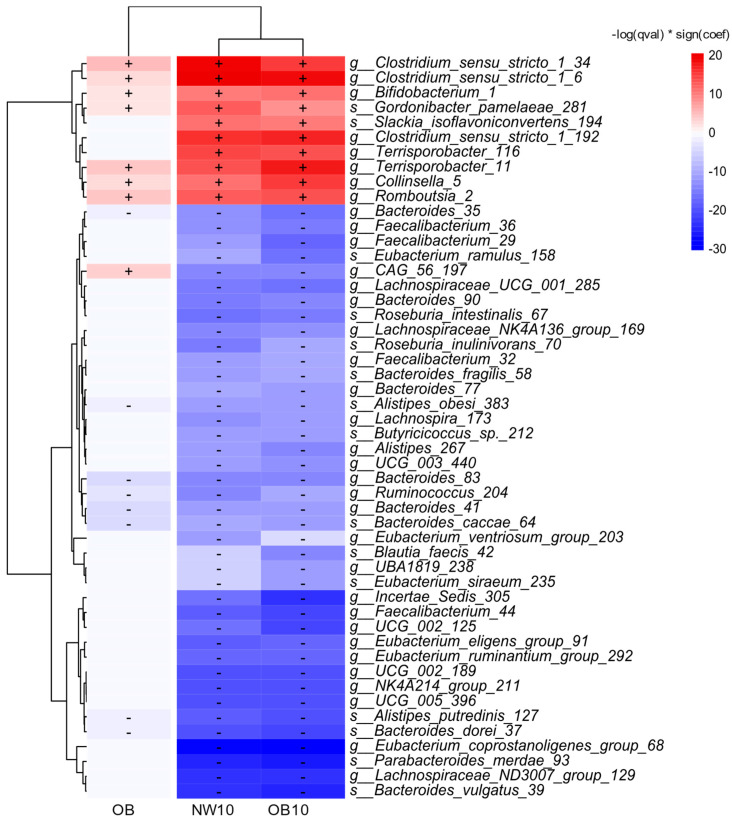
A heatmap displaying the top 50 ASVs with significant changes in relative abundance between the study groups and NW group (used as the reference), detected by MaAsLin2s with default parameters. An increase in relative abundance is shown in red (+) and a decrease in this is shown in blue (−).

We examined the taxa significantly associated with BMI and BPA exposure using a linear mixed model (MaAsLin2), with the reference group defined as NW. The results are shown in Figure 3. ASVs positively associated with the OB group and BPA-exposed groups (NW10, OB10) belonged to the genera *Clostridium sensu stricto* 1, *Bifidobacterium*, *Collinsella*, *Romboutsia*, and *Terrisporobacter*.

In contrast, negatively associated genera included *Alistipes*, *Bacteroides*, *Eubacterium*, and *Ruminococcus*. Notably, several of the top 50 significantly different ASVs, which were linked to the OB group, also showed associations with the BPA-exposed groups, suggesting that obesity triggers changes in the microbiota that are similar to the effects of exposure to BPA.

### 3.3. Culturing of Human Gut Microbiota: Effect of BPA Xenobiotics on Isolation of Bacteria

#### 3.3.1. Microbiological Count

**Figure 4:**
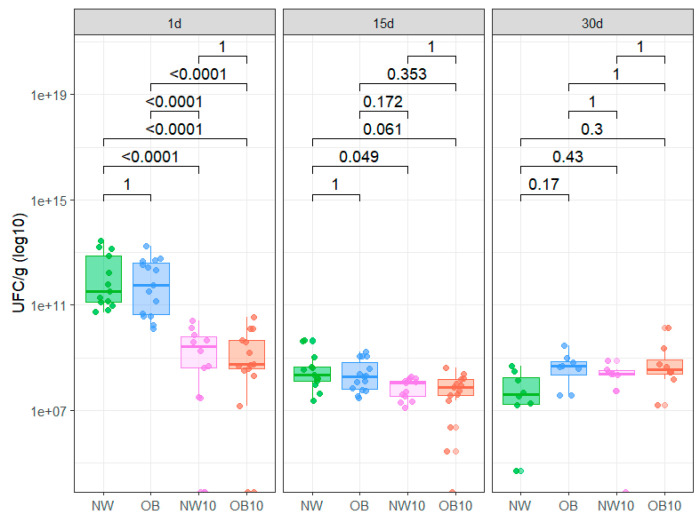
Box plot representing CFU/g from each study group after 1, 15, and 30 days of anaerobic culture in recovery enrichment media. NW, green; OB, blue; NW10, pink; OB10, orange.

The culture assay results from CFU/g counts after 1, 15 and 30 days under enrichment conditions are displayed in Figure 4. We did not find significant differences in CFU/g regarding BMI groups. However, we observed a significant decrease in CFU/g in BPA-exposed samples after 1 day of incubation compared to non-exposed samples. Similarly, at 15 days, the CFU/g counts in NW10 and OB10 samples were also decreased, though not significantly. In contrast, after 30 days of culture enrichment, this trend slightly reversed, showing the recovery of microbial colonies, indicating the potential retrieval of spore-forming bacteria in all study groups.

#### 3.3.2. Identification of Cultured Isolates

**Figure 5:**
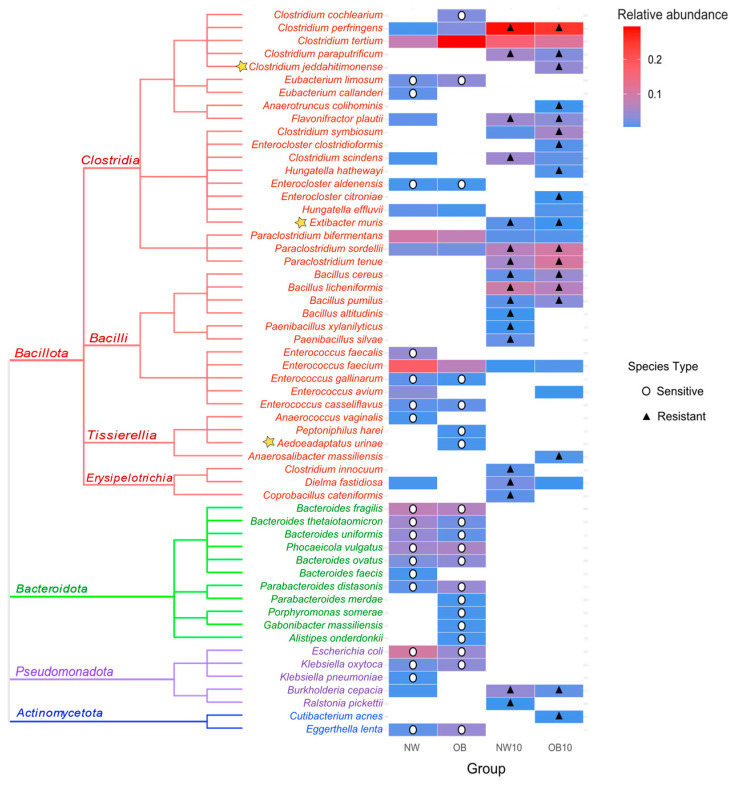
Heatmap of the relative abundance of identified isolates in each group, accompanied by a phylogenetic tree. Stars highlight species identified by sequencing after MALDI TOF failed to identify them. Circles indicate species sensitive to BPA, cultured in control samples (NW, OB) but absent after being exposed to BPA (NW10, OB10). Triangles indicate species showing resistance to BPA, only cultured after exposure to BPA or those whose relative abundance was at least double that of non-exposed samples.

The described culturing methodology enabled the isolation and identification of 655 bacteria, of which 144 were from the NW group, 139 from the OB group, 200 from NW10 group, and 172 from the OB10 group. A total of 56 different viable species were isolated, 53 of which were identified by MALDI-TOF and 3 of which were taxonomically annotated by the sequencing of the 16S rRNA gene. The relative abundance of the isolated species was calculated and represented in a heatmap accompanied by a phylogenetic tree (Figure 5). The notable variability in species from the phylum *Bacteroidota* was observed, with a high abundance in both non-exposed groups (NW, OB). These species were not isolated after BPA exposure, so they have been classified as sensitive to BPA (indicated by white circles). Similarly, species belonging to *Enterococcus* and *Eubacterium* genera were also not found after BPA exposure.

In contrast, several species that were either not isolated or were present in low abundance in non-exposed samples appeared in high abundance in the BPA-exposed samples (NW10, OB10). These species were considered and classified as resistant to BPA (indicated by black triangles). Most of these resistant species belong to the *Bacillota* phylum, predominantly classified within the *Clostridia* class or *Bacilliales* order (*Bacillus* and *Paenibacillus* genera). We also noted the resistance of interesting species from the microbiota such as *Flavonifractor plautii*, *Extibacter muris*, *Paraclostridium* spp., and *Burkholderia cepacia*.

### 3.4. Taxa Comparative Analysis of 16S rRNA Amplicon Sequencing and Culturing Data

**Figure 6:**
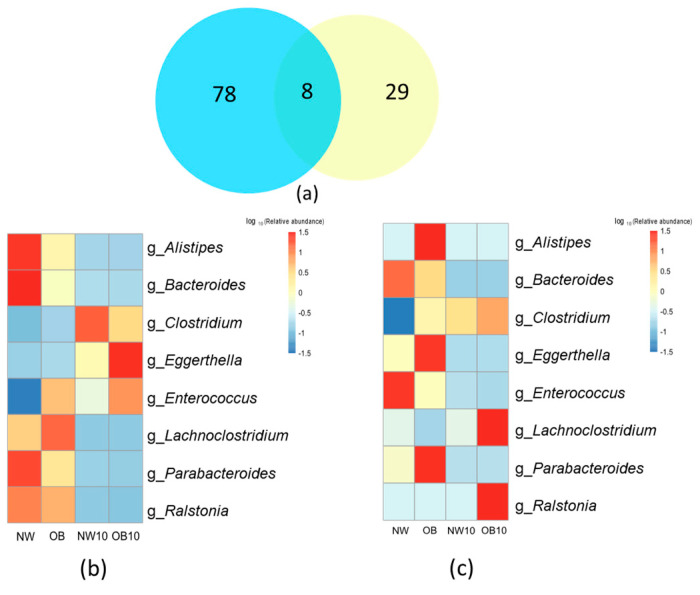
Comparison of 16S rRNA sequencing and culturing results. (**a**) Venn diagram depicting overlap between genera identified by 16S rRNA sequencing (blue) and culturing (yellow); (**b**,**c**) heatmaps representing the relative abundance of the common genera identified by 16S rRNA sequencing and culturing. “g_*Clostridium*” represents the relative abundance of “g_*Clostridium sensu stricto* 1”.

After comparing the 16S rRNA gene sequencing and culturing results at the genus level, only eight genera were shared between both methodologies (Figure 6a).

The loss of the *Bacteroides* genus after exposure to BPA was observed in the results of the two strategies applied here (Figure 6b,c). The relative abundance of this genus was significantly reduced and was associated with low the BMI in the non-BPA-exposed group (NW) on sequencing analysis. In addition, all cultivable species from the *Bacteroides* genus were classified as sensitive to BPA in the culturomics approach.

However, other taxa were only identified through sequencing, such as *Bifidobacterium*, *Prevotella*, as were other genera of interest (Figure 6b). The culture methodology did not recover the growth of these genera in either the control groups (NW, OB) or the BPA-exposed groups (NW10, OB10). Conversely, species that were promoted in cultures, such as several viable species belonging to the *Bacilli* class found in BPA-exposed samples, were not well represented in the 16S rRNA sequencing relative abundance results.

### 3.5. Validation of BPA-Induced Changes in Microbiota and Their Association with Obesity

**Figure 7:**
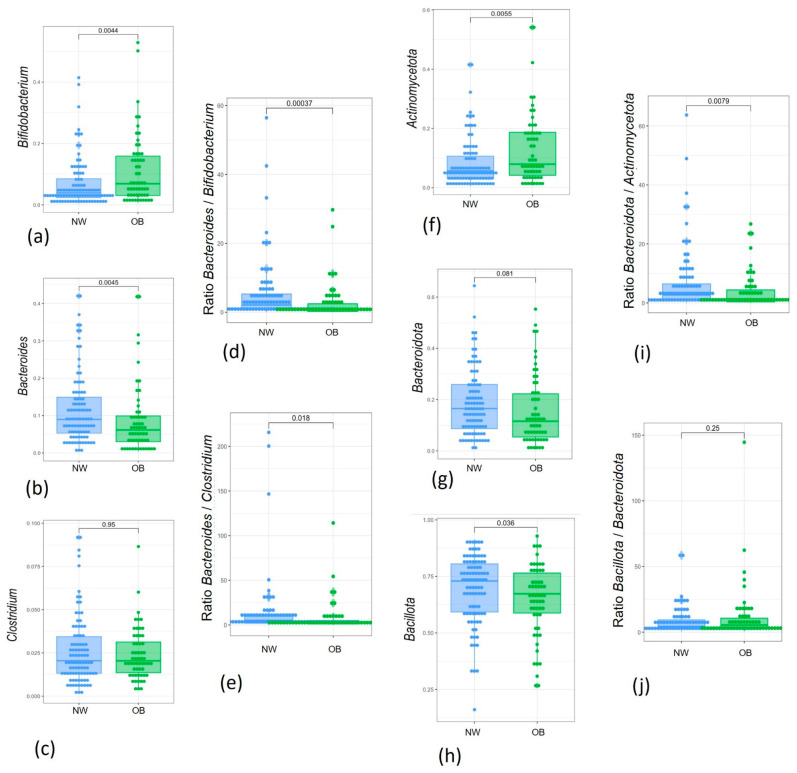
Box plots showing the relative abundance distribution of significantly different bacterial genera and phyla and their specific ratios between study groups: (**a**) *Bifidobacterium*; (**b**) *Bacteroides*; (**c**) *Clostridium*; (**d**) *Bacteroides*/*Bifidobacterium* ratio; (**e**) *Bacteroides*/*Clostridium* ratio; (**f**) *Actinomycetota*; (**g**) *Bacteroidota*; (**h**) *Bacillota*; (**i**) *Bacteroidota*/*Actinomycetota* ratio; (**j**) *Bacteroidota/Bacillota* ratio. NW, green; OB, blue.

**Figure A2:**
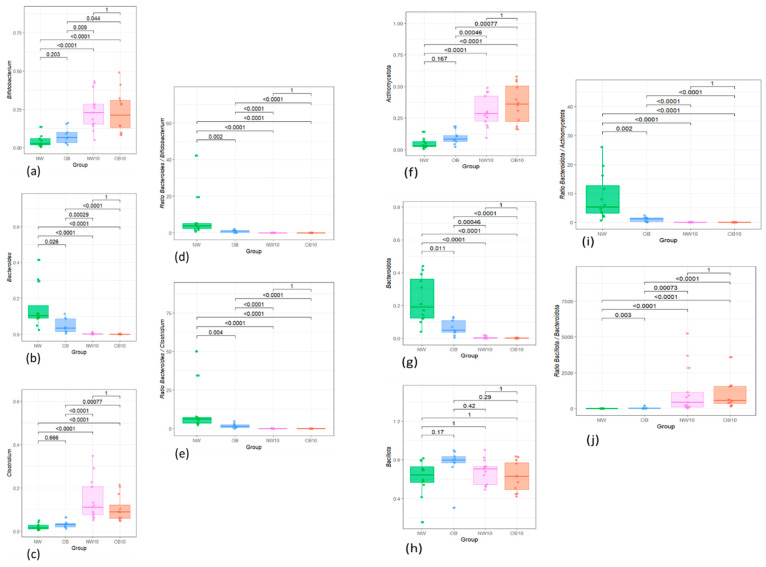
Boxplots showing the relative abundance distribution of significantly different bacterial genera and phyla and their specific ratios between study groups (**a**) *Bifidobacterium*; (**b**) *Bacteroides*; (**c**) *Clostridium*; (**d**) Ratio *Bacteroides*/*Bifidobacterium*; (**e**) Ratio *Bacteroides*/*Clostridium*; (**f**) *Actinomycetota*; (**g**) *Bacteroidota*; (**h**) *Bacillota*; (**i**) Ratio *Bacteroidota*/*Actinomycetota*; (**j**) Ratio *Bacteroidota/Bacillota*. NW, green; OB, blue; NW10, pink; OB10, orange.

The patterns of specific bacterial taxa observed in this pilot study (Figure A2) were subsequently validated in a larger project (Figure 7) and set of samples from the same cohort of children, data derived from López et al. 2024 [15] (Supplementary Materials). 

The relative abundance of *Bifidobacterium*, which increased after BPA exposure, was also higher in the OB group compared to the NW group in our pilot study, although this difference was not significant (Figure A2a). However, this comparison became significant in the larger cohort, with its greater statistical power (Figure 7a). Moreover, the reduction in *Bacteroides* genus relative abundance observed by the two strategies applied in this pilot study was also verified in the cohort of 150 children when comparing samples from children with obesity to normal-weight children (Figure 7b). In contrast, the increase in *Clostridium sensu stricto* 1 *Clostridium* genus) observed after BPA exposure in both approaches of our pilot study was not significant in the OB group compared to the NW group, even after validation in the 150 samples.

Several taxa ratios influenced by these patterns that were associated with BPA exposure and obesity dysbiosis were of interest when comparing the study groups. In the larger cohort analysis, the *Bacteroides/Bifidobacterium* ratio was significantly different between OB and NW groups (Figure 7c), which may validate previous microbiota patterns found after BPA ex vivo exposure. Analysis of the 150 samples also revealed differences in the *Bacteroides/Clostidium* ratio (Figure 7d).

A similar analysis was conducted at the phylum level. *Actinomycetota* was significantly higher in exposed groups (Figure A2f) and increased in the OB group. This pattern was confirmed in the larger cohort, comparing the OB group with the NW group (Figure 7f). In contrast, the relative abundance of *Bacteroidota*, which decreased in both the OB and BPA-exposed groups (NW10, OB10), was borderline significant in the larger sample analysis (Figure 7g). Finally, while *Bacillota* did not show changes in the pilot study, the results from the 150-sample cohort indicated a significant decrease in the OB group.

Regarding the *Bacteroidota*/*Bacillota* ratio, no significant difference was found between obese and normal-weight samples (Figure 7j). However, the new ratio of *Bacteroidota/Actomycetota* was significantly reduced in children with obesity (Figure 7i).

## 4. Discussion

The remarkable findings of this study provide valuable insights into the complex interplay between the BPA–obesity–microbiota triad. Our results highlight the relevance of xenobiotics in influencing the composition and structure of the microbiota, and its relationship with the obesity-associated microbial profiles. It is well known that dietary habits also determine obesity phenotypes. In this regard, a recent study has explored the impact of diet and bisphenol exposure, examining BMI, energy intake, and Mediterranean dietary scores in a larger Spanish child cohort using validated questionnaires [31]. The authors disclosed valuable data linking dietary BPA intake in this population to obesogenic effects, incorporating differential gender results. Further data considering microbiome analysis will complement these outcomes, as it is well recognised that gut microbiota plays a vital role in obesity and human health. Interestingly, it possesses microbial enzymes and key pathways capable of metabolising and degrading xenobiotics. The impact of some xenobiotics on human health and the microbiota has been previously reviewed [32]. Changes in the microbiota composition, such as dysbiosis caused by exposure to BPA, appeared to contribute to inflammation in the gut and develop several diseases [33,34]. Such alterations could subsequently impact key metabolic and immunological functions in which these microorganisms or their metabolites are involved [14]. In consequence, BPA affects microbiota composition and, ultimately, human health, promoting metabolic disorders [35,36] and body composition modifications linked to obesity [37].

In our study, data from 16S rRNA amplicon sequencing and culture analyses revealed that BPA-exposed groups experienced a loss of microbiota species richness, selecting for a community capable of adapting to the presence of the xenobiotic. Additionally, results showed that the microbiota composition and structure of children with obesity were more similar to those of BPA-exposed groups than those of normal-weight children. This suggests that xenobiotic exposure could induce a form of dysbiosis comparable to that observed in childhood obesity. Similar findings were previously reported by Lai et al. (2016), where mice exposed to BPA or fed a high-fat diet exhibited reduced alpha diversity and equivalent changes in gut microbiota composition [38].

Our most notable finding from the complementary methodologies used was the depletion of species belonging to the *Bacteroidota* phylum, specifically to the genus *Bacteroides*, in both BPA-exposed groups. Similarly, *Bacteroides* loss after BPA exposure was corroborated by several animal studies [39,40,41,42,43,44,45,46,47,48]. This reduction may have critical implications for animal and human health, given the role of *Bacteroides* in maintaining intestinal barrier integrity [49], regulating inflammatory pathways [50], and supporting metabolic balance. Moreover, Hong et al. (2022, 2023) linked *Bacteroides* depletion to weight gain, fat accumulation, and altered lipid profiles in murine models [41,51]. A potential mechanism by which BPA affects *Bacteroides* species may involve its hydrophobic properties, which could lead to membrane destabilisation. Riesbeck et al. (2022) demonstrated BPA’s impact on *Bacteroides thetaiotaomicron* through the reduction in membrane fluidity, together with an alteration in short-chain fatty acid production and a disruption of energy metabolism [52]. Several studies also showed that bisphenols can aggregate within bacterial membranes, causing lipid desorption, pore formation, and altered membrane integrity [53]. Interestingly, this effect seemed to be specific to bisphenols, as other xenobiotics, such as chlorpyrifos, increased *Bacteroides* abundance while decreasing *Bifidobacterium* [54].

BPA promoted the abundance of genera belonging to the *Actinomycetota* phylum, including the genera *Bifidobacterium* and *Collinsella,* according to 16S rRNA amplicon sequencing results. Although the culturing approach effectively identified diverse microbiota species, these genera were not recovered as cultured isolates, suggesting that more specific growth media for these genera should be used [55,56]. Previous studies reported an increase in *Collinsella aerofaciens* and *Bifidobacterium adolescentis* when a model of the gut microbial community was exposed to BPA and other molecules from the plastic industry [57]. Some species of the genus *Bifidobacterium* could proliferate in a xenobiotic-rich environment, a phenomenon potentially linked to their ability to sequester BPA in vitro [58]. This capacity for bioaccumulation not only enables these bacteria to thrive but also reduces BPA concentrations in the surrounding medium. Additionally, Shoukat et al. (2019) observed that *Bifidobacterium* could degrade the xenobiotic benzo[a]pyren, with the binding of this molecule to the bacterial cell wall being a key factor in the degradation mechanism [59].

The culturomic approach allowed the isolation of several species classified as BPA-resistant, but they were not observed in the 16S rRNA amplicon sequencing approach. In this regard, this methodology was therefore essential to isolate BPA-resistant species and to study the potential microbial biodegradation of BPA and its role in mitigating toxicity, as previously described [15]. In our study, species belonging to the *Bacilli* and *Clostridia* classes were long-term viable and potential BPA-degrading species, probably because they are endospore-forming bacteria, also called sporobiota [60,61]. In addition, several *Bacillus* species presented enzymatic BPA biotransformation capacities [62,63,64]. Such degradative activity could be harnessed to reduce the harmful effect of BPA in vivo. Other potentially BPA-resistant isolated species, such as *Flavonifractor plautii* and *Extibacter muris*, might become of great interest in future BPA biodegradation studies due to their associated health benefits [65,66,67].

In summary, we observed concordance in microbial patterns after BPA exposure and those related to obesity. The consistent decrease in *Bacteroides* and the increase in *Bifidobacterium* observed in obesity and BPA-exposed samples may indicate a common profile of dysbiosis. This altered pattern may influence human metabolic and physiological states, with the microbiota potentially acting as an intermediary in this process. Moreover, this effect is supported by the recent evidence identifying BPA as an obesogenic xenobiotic [68]. A low abundance of *Bacteroides* has also previously been associated with obesity [69]. However, unlike what our findings suggest, *Bifidobacterium* is typically reduced in obesity, according to earlier studies [70,71]. This discrepancy may be attributed to the age of the participants, as other studies reported a higher abundance of *Bifidobacterium* in children, which tends to decrease with ageing [63,72].

The *Bacteroides*/*Bifidobacterium* ratio could be useful for discriminating between microbiota samples from children with obesity or normal-weight children. Other similar ratios, such as those of *Prevotella*/*Bacteroides* [73,74] or *Blautia/Bacteroides* [75], have been proposed in the literature related to weight alterations. Conversely, the *Bacteroides*/*Bifidobacterium* ratio is rarely described in relation to obesity phenotypes. This may be due to the complexity of the interactions between bacteria, and factors involved in the development of obesity. For instance, *Bacteroides* species provide intermediary metabolites that can be utilised by other bacteria, such as *Bifidobacterium* [76]. However, the interaction between both genera is not always mutualistic, as they can compete with each other when they are cultivated with specific carbon sources [77]. The presence of BPA in the intestine is another disrupting factor that may affect the microbiota and its metabolites, further complicating the relationships between their consortia organisations. A recent study demonstrated that pollution and xenobiotic exposure also disrupted human gut microbiota. In line with our data, the authors observed an increased abundance of *Bifidobacteria*, and *Clostridium,* among other species, and a decrease in *Bacteroides* in areas with high pollution [78]. These findings align with those of previous research, which emphasises the importance of understanding interactions in shaping microbial community organisation, enzymatic capacities against xenobiotic exposure, and metabolic outcomes [79].

Importantly, at the phylum level, an increase in *Actinomycetota* in our Spanish cohort of obese children was observed, which is in line with recent results from a Chinese obese cohort [80]. In addition, the *Bacteroidota*/*Actinomycetota* ratio showed remarkable and significant differences when comparing normal-weight children with children with obesity. In contrast, the *Bacillota*/*Bacteroidota* ratio did not show significant differences. Despite being a well-recognised ratio, it has also shown discrepancies in phenotypic outcomes in the scientific literature [81]. Therefore, we suggest that new microbiota ratios based on the findings of our microbiome metrics and validated tests could serve to identify useful potential biomarkers related to altered biological status. Moreover, these ratios should be viewed as preliminary data for interpreting potential clinical effects and related interactions between microbiota, xenobiotics, and childhood obesity.

A potential limitation of this study is the small sample size used for the pilot assay. However, the complementary and combined approaches applied together with a larger cohort for further validation overcome this issue. Culturomics using tailor-made growth media for key differential bacteria will increase the effectiveness of taxa similarity data comparisons. Short-term, ex vivo BPA exposure may not fully capture the chronic long-term effects on microbiota composition and human health, which are beyond the scope of this study.

## 5. Conclusions

BPA-susceptible and resistant bacteria were identified and reliably confirmed through various analytical approaches and complementary experimental methodologies, providing the basis for developing new strategies for BPA degradation in future studies under a One-Health approach. This research shows the link between BPA exposure and obesity mediated by common altered patterns of microbiota taxa. Based on this, novel microbiota ratios such as those of *Bacteroidota*/*Actinomycetota* and *Bacteroides*/*Bifidobacterium* are proposed as potential biomarkers to evaluate microbiota dysbiosis triggered by BPA and obesity. However, further biomonitoring studies and meta-analyses will be needed to validate these findings.

## Figures and Tables

**Table 1:** Anthropometric characteristics of participants.

Variable	NW (n = 15)	OB (n = 13)	*p* Value
**Gender**			1
Male	6 (40%)	6 (46%)	
Female	9(60%)	7 (53.85%)	
**Age**			0.112
Median (IQR)	8 (2.50)	8 (2.00)	
Range	5–10	6–11	
**BMI** ^1^			<0.001 *
Median (IQR)	15.43 (2.36)	23.47 (4.11)	
Range	13.1618.04	19.47–27.081	

^1^ BMI: body mass index; NW: normal weight; OB: obese. * Significant *p* value.

## Data Availability

The original contributions presented in this study are included in the article/Supplementary Material. Further inquiries can be directed to the corresponding author.

## Supplementary Materials

The following supporting information can be downloaded at https://www.ncbi.nlm.nih.gov/bioproject/PRJNA979040. ASV sequences for validation in the microbiota of the cohort of children are deposited in the NCBI Sequence Read Archive (SRA) database with the Bioproject number PRJNA979040.

## Appendix A

**Table A1:** Result of PERMANOVA pairwise analysis.

Pairs	R^2^	*p* Value	FDR
OB vs. NW	0.06220594	0.195	1
OB vs. NW10	0.22548776	0.001	0.006
OB vs. OB10	0.25366861	0.001	0.006
NW vs. NW10	0.29545063	0.001	0.006
NW vs. OB10	0.31864552	0.001	0.006
NW10 vs. OB10	0.05037656	0.237	1

## Appendix B

Protocol for Bovine Rumen Fluid Extraction:

1. Collect and place all possible rumen contents into a container, ensuring it is sealed tightly to minimise air exposure.
2. Drain the solid matter and pass the liquid through a nylon or plastic sieve.
3. Filter the obtained liquid again using a mesh.
4. Centrifuge the liquid at 8000 rpm for 20 min using 50 mL tubes and collect the supernatant.
5. Repeat the centrifugation process 5 times.
6. Filter the supernatant sequentially through the following filters: 0.8 µm, 0.45 µm and 0.22 µm.
7. After the last filtering, the liquid is sterile and ready for use in microbiological culture experiments.
